# Pathogenesis of thoracic ossification of the ligamentum flavum

**DOI:** 10.3389/fphar.2024.1496297

**Published:** 2024-10-31

**Authors:** Chi Zhang, Yanan Chang, Li Shu, Zhongqiang Chen

**Affiliations:** ^1^ Department of Orthopedics, Peking University International Hospital, Beijing, China; ^2^ Central Laboratory, Peking University International Hospital, Beijing, China; ^3^ Biomedical Engineering Department, Institute of Advanced Clinical Medicine, Peking University, Beijing, China

**Keywords:** thoracic ossification of the ligamentum flavum (TOLF), bone formation, ossification, pathogenesis, OSX, osteoporosis

## Abstract

Thoracic ossification of the ligamentum flavum (TOLF) is characterized by ectopic ossification of the ligamentum flavum in the thoracic spine and is considered the main cause of thoracic spinal stenosis and spinal cord disease. Osteoblast specific transcription factor Osterix (Osx) is required for bone formation, and there is no bone formation or ossification without Osx. Surgical intervention is recognized as the only effective method for TOLF treatment with set of complications. However, underlying mechanisms of TOLF are not well understood. This paper summarizes the pathogenesis of TOLF. Some relevant factors have been discussed, such as mechanical stress, genetic susceptibility genes, endocrine and trace element metabolism abnormalities, which may associate with TOLF. More recent studies using proteomics technology and RNA sequencing approach have discovered that some new factors participate in TOLF by upregulation of Osx gene expression including inflammatory factors. TOLF is a unique disease involving multiple factors. On the other hand, studies on TOLF pathogenic mechanism may provide new ideas for finding possible upstream regulatory factors of Osx and further developing novel drugs to stimulate new bone formation to treat osteoporosis.

## 1 Introduction

Heterotopic ossification refers to the appearance of bone tissue in areas where bone formation should not normally occur. This phenomenon is usually seen in soft tissues, which are regions that do not typically contain bone, such as muscle, ligament, tendon, or almost any site of connective tissue. Heterotopic ossification is a common complication of spinal cord injury (Cao et al., 2023). Ossification of the ligamentum flavum (OLF) is a type of ectopic ossification disease of the spinal ligaments, characterized by the transformation of the fibrous tissue within the ligament into bony tissue. OLF can occur in the cervical, thoracic, and lumbar regions of the spine, with the thoracic region, particularly the lower thoracic segment (T9∼T12), being the most common site of occurrence (Guo et al., 2010; Lang et al., 2013; Hur et al., 2009).

Thoracic ossification of the ligamentum flavum (TOLF) is characterized by ectopic ossification of the ligamentum flavum in the thoracic spine and is considered the main cause of thoracic spinal stenosis and spinal cord disease. The onset of TOLF is insidious, and there are no obvious symptoms when the spinal cord is not compressed in the early stage. However, slow and progressive compression of the thoracic spinal cord nerves can cause serious consequences such as sensorimotor disorders, bowel and bladder dysfunction, and even paraplegia (Liang et al., 2019). However, underlying mechanisms of TOLF are not well understood.

There are two forms of bone formation: intramembranous osteogenesis and endochondral osteogenesis. Intramembranous osteogenesis involves the differentiation of mesenchymal cells into an embryonic connective tissue membrane, which then forms bone within the membrane. This process occurs in the craniofacial and clavicle regions (Compston et al., 2019); Osteogenesis within cartilage is characterized by the process of pre-formed cartilage serving as a template and being replaced by bone, which is much more complex than intramembranous ossification. Most bones, such as limbs, trunk, and skull base, occur in this way (St-Jacques et al., 1999). Osteoblasts differentiate from mesenchymal stem cells and undergo multiple stages controlled by different transcription factors. Ihh is an essential transcription factor for endochondral osteogenesis and does not participate in intramembranous osteogenesis (St-Jacques et al., 1999). It is indispensable for the differentiation of mesenchymal cells into osteoblasts; The transcription factor Runx2 is involved in the regulation of endochondral and intramembranous osteogenesis, and cells expressing Runx2 can differentiate into osteoblasts or chondrocytes (Komori et al., 1997); Osteoblast specific transcription factor Osterix (Osx) is an essential transcription factor for bone formation and osteoblast differentiation (Nakashima et al., 2002). Embryos of OSX gene deficient mice have no bone formation and cartilage formation is not affected. Our research group has identified some important downstream target genes of OSX, including VDR, SATB2, VEGF, DKK1, SOST, *etc.*, confirming the importance of OSX in bone formation (Zhang, 2012). OSX also regulates the osteoblast marker gene bone sialoprotein directly (Yang et al., 2016).

On the contrary, osteoporosis is the most common metabolic bone disease characterized by reduced bone mass, microstructural changes in bone tissue and decreased bone strength. Osteoporosis is caused by the functional imbalance between osteoblasts and osteoclasts. At present, most osteoporosis treatment drugs aim to inhibit bone resorption, and there is no drug that can specifically stimulate new bone formation directly. Elucidating the gene regulatory molecular mechanisms of bone formation is of great significance for guiding the development of new drugs for osteoporosis to specifically promote bone formation. There is no bone formation or ossification without Osx. Genome wide association analysis studies have confirmed that OSX is associated with osteoporosis (Timpson et al., 2009). Although OSX is associated with the phenotype of osteoporosis, further research is needed to determine the molecular mechanisms of OSX in bone formation, including exploring upstream regulatory factors of Osx. The treatment of osteoporosis urgently requires new drugs for bone formation. Thoracic ossification of the ligamentum flavum is a unique bone disease. Due to unknown pathological reasons, TOLF stimulates the formation of new bone in the ligament flavum, which might provide a great reference for searching for upstream regulatory factors of Osx.

## 2 Diagnosis and classification of TOLF

Imaging examination is the most commonly used method for the localization and qualitative diagnosis of TOLF (Hirabayashi, 2017). Thoracic vertebrae lateral X-ray film is the basic initial screening method for TOLF, but due to the influence of the occlusion of structures such as the shoulder or liver, the qualitative and positional diagnostic role of X-ray for TOLF is limited, and its clinical application value is low. At present, CT and MRI play important roles in the imaging diagnosis of TOLF, which can respectively display the morphology, position, ossification, and nerve compression of TOLF in the axial and sagittal positions. Among them, CT can better reflect the degree of ossification, while MRI can better reflect the spinal nerve compression. The combination of the two is conducive to the precise diagnosis of TOLF (Takahashi et al., 2018; Yu et al., 2013).

There are numerous TOLF classification systems clinically established based on ossification morphology, distribution, spinal cord compression degree, and the relationship with surrounding tissues. Among them, Sato classification (lateral type, extensive type, enlarged type, fusion type, nodular type) based on CT (Sato et al., 1998) and Kuh classification (beak type, round type) based on MRI (Kuh et al., 2006) are more commonly used clinically. Other scholars' classifications such as Chen classification (focal type, skip type, continuous type) and Muthukumar classification (Tram track sign, Comma sign) (Muthukumar, 2009) with ossification of the dural sac have expounded on the types of TOLF from different angles and dimensions, with certain clinical value in reflecting ossification mechanisms, assessing disease risks, guiding treatment plans, or judging prognosis. These classification systems are of high accuracy in diagnosis and reliability in classification. Table 1 summarizes various classification systems that have been reported currently.

**TABLE 1:** The classification systems of TOLF.

Classification basis	Classification system	Type	Imaging findings
X-ray	Kudo classification Based on the morphology of ossification	Hook	Ossification grows from the upper or lower edge of the vertebral lamina, with the tip pointing to the intervertebral foramen
		Beak	Ossification grows simultaneously from the upper and lower edges of the vertebral lamina to the middle, with the tip pointing to the intervertebral foramen, resembling a beak
		Linear	Thin ossification is limited to the upper edge or upper and lower edges of the vertebral lamina
		Nodular	Ossification is located at the posterior margin of the intervertebral foramen in nodular shape
CT	Okada Classification Based on the morphology of ossification nests on CT cross-sectional images	Lateral	Ossification can be found in the articular capsule part of the ligamentum flavum
		Diffuse	Ossification can be observed in the superficial layer of the hypertrophied ligamentum flavum, where the laminar bone is dense but the medullary cavity is underdeveloped. Additionally, the cortical thickening of the vertebral lamina and significant stenosis of the thoracic spinal canal are present
		Thickened Nodular	The cephalic part of the ossification shows a nodular shape, while the caudal part exhibits a thickened form, extending to the capsular and pedicular portions. It protrudes into the spinal canal in the form of a beak-like protrusion
	Sato Classification Based on the growth direction of ossification on CT cross-sectional images	Lateral	Ossification is confined to the articular capsule portion of the ligamentum flavum, detectable at the lateral margin of the spinal canal
		Extended	Ossification extends to the intervertebral laminae portion of the ligamentum flavum, but the ossification is thin and has not yet invaded the spinal canal
		Enlarged	The ossification thickens anteromedially towards the spinal canal, but the midlines on both sides do not fuse
		Fused	Ossification fuses at the midline of the vertebral lamina, forming a “V” shape
		Tuberous	Ossification protrudes centrally in a nodular shape, invading the spinal canal
	Kuh Classification Based on the location and morphology of ossification	Unilateral	Unilateral ossification of the ligamentum flavum
		Bilateral	Bilateral ossification of the ligamentum flavum, but without fusion between the two sides
		Bilateral	Bilateral ossification of the ligamentum flavum, with fusion between the two sides forming a bridge-like plate
MRI	Kuh Classification Based on the morphological Characteristics of ossification observed on T2-weighted sagittal MRI	Beak	The ossification resembles a “beak” protruding forward, causing localized compression of the spinal cord
		Round	The anterior end of the ossification exhibits an arcuate protrusion, leading to diffuse compression of the spinal cord
	Chen classification Based on the distribution of ossification	Isolated	Ossification is localized between two vertebral segments
		Skipping	Ossification is discontinuously distributed, with focal and continuous types potentially coexisting
		Continuous	Ossification continuously affects three or more vertebral segments
	Moon classification Based on the degree of ossification invading the spinal canal	Grade 0	no cord compression
		Grade 1	minimal subarachnoid space compression on the midline image or moderate subarachnoid space compression on the offset image
		Grade 2	mild compression on the midline image
		Grade 3	moderate cord compression or cord signal change
	Yamashita Classification Based on the signal intensity of TOLF on T2-weighted imaging	No Signal	Ossified region appears as black signal
		Low Signal	Lower than normal spinal cord signal
		Iso-Signal	Same as normal spinal cord signal
		High Signal	Higher than normal spinal cord signal
Associated with dural ossification	Muthukumar classification Based on the characteristics of TOLF combined with dural ossification	Tram Track Sign	There is a low-density gap between the high-density dural ossification and TOLF, shaped like a track
		Comma Sign	The fused portion of dural ossification combined with TOLF constitutes the tail end of a “comma”-shaped configuration

Note: This table summarizes various classification systems that have been reported currently.

The ligamentum flavum is located at the posterolateral side of the vertebral canal of the spine, connecting two adjacent vertebral plates. It is divided into two parts: the capsular and the interlaminar portions. It consists of 80% elastic fibers and 20% collagen fibers (Yong-Hing et al., 1976). Ossification of the ligamentum flavum is more likely a process of endochondral ossification (Yayama et al., 2007; Nishikawa et al., 2023). The pathological changes initiate from the dural surface, characterized by early disruption of fiber arrangement, degeneration and degradation of elastic fibers, along with proliferation, swelling, and mucoid degeneration of collagen fibers. Meanwhile, local fibroblasts undergo chondrogenic differentiation into fibrochondrocytes, secreting chondromatrix to form cartilage. Subsequently, the cartilage undergoes calcification, with neovascularization invading the cartilage, which induces local mesenchymal cells to differentiate into osteoblasts. These osteoblasts then secrete bone matrix to form mature bone. Ossified ligamentum flavum typically exhibits four transitional zones from superficial to deep: ligament zone, chondroid zone, calcified cartilage zone, and ossified zone.

## 3 Epidemiology of TOLF

Based on previous studies, TOLF mainly occurs in Asian populations in China, Japan, South Korea and other countries. The prevalence of TOLF is 3.8%–63.9% in Chinese (Guo et al., 2010; Lang et al., 2013; Liang et al., 2019), 3.6%–36% in Japanese (Fujimori et al., 2016; Mori et al., 2013; Ohtsuka et al., 1986), and 16.9%–21.8% in Koreans (Kim et al., 2018; Moon et al., 2015). Meanwhile, TOLF is more common in the elderly population. One report has shown that the average age of onset is 61 years old. When Asians are over 65 years old, the incidence can be up to 20% (Aizawa et al., 2006). However, there are few reports on the incidence of TOLF in European, American and African populations (Pascal-Moussellard et al., 2005; Ricciardi et al., 2021). Due to the limitation of the number of studies and sample sizes, it is difficult to compare the prevalence of TOLF among Asian, European and American populations.

## 4 Therapeutic method of TOLF

TOLF is a static compressive disease with insidious onset, and its clinical symptoms may progress as ossification of ligamentum flavum extends. Furthermore, TOLF frequently coexists with other spinal diseases, such as intervertebral disc herniation or ossification of the posterior longitudinal ligament. The lesion distribution can occur from a single segment to the entire thoracic spine, and it may lead to complex and diverse clinical manifestations that are prone to misdiagnosis and missed diagnosis (Yamada et al., 2021). Once TOLF compresses the thoracic spinal cord, it causes severe damage to motor neurological function, and the clinical symptoms are often severe, with an extremely high rate of paralysis (Feng et al., 2015).

Surgical intervention is widely recognized as the only effective method currently available for the treatment of TOLF. The aim of surgical intervention is to remove the ossified segments and provide adequate decompression. Posterior decompression surgery is the most common surgical approach, and patients often experience significant improvement in symptoms after the procedure. Various surgical techniques have been reported, including open-door laminectomy, *en bloc* laminectomy, hemilaminectomy, and fenestration, each with different decompression ranges (Kuh et al., 2006). A consensus has generally emerged that the resection should include the laminae, the medial 1/3 to 1/2 of the facet joints, the thickened and ossified ligamentum flavum, as well as one or two layers above and below the affected segments. In TOLF patients with dural ossification, the ossified dura mater and OLF are resected *en bloc* with preserving arachnoid (Hirabayashi, 2017).

In recent years, with significant advancements in surgical techniques, clinicians have obtained abundant and precise preoperative information through CT and MRI imaging, and intraoperative imaging modalities such as navigation systems have been introduced, making decompression surgery safer (Hirabayashi, 2017). Some scholars have explored the use of endoscopic techniques for the resection of TOLF. Yang et al. performed an endoscopic full-spine surgery, utilizing matched ultrasonic osteotomy to directly remove single-level TOLF, achieving satisfactory surgical outcomes (Yang et al., 2021). Bagga et al. conducted a retrospective cohort study and found that compared to conventional high-speed burr resection of TOLF, the use of ultrasonic osteotomy significantly shortened the operative time, reduced dural tears, and increased the safety and efficacy of decompression surgery (Bagga et al., 2023). Liu et al. utilized percutaneous endoscopic thoracic decompression (PETD) to treat multi-level TOLF, also achieving favorable results with symptom relief (Liu et al., 2023). This demonstrates the significant advantages of spinal endoscopic techniques in TOLF treatment. However, endoscopic thoracic surgery poses technical challenges and requires experienced surgeons, as it may be accompanied by serious complications (Park et al., 2023).

Every surgical procedure carries its unique set of complications. Common surgical complications associated with TOLF include spinal cord or nerve root injury, dura mater injury with cerebrospinal fluid (CSF) leakage, epidural hematoma formation, postoperative spinal instability, and even kyphosis. Among these, dura mater injury and CSF leakage are the most prevalent (Osman et al., 2018), primarily resulting from severe ossification causing compression of the dura mater, leading to its adhesion and even ossification. Zhang et al. (2023) reported in their study of 266 TOLF patients that the incidence of dura mater injury combined with CSF leakage was 32%, causing discomfort to patients. Through a series of comprehensive treatments such as intraoperative dura mater repair, postoperative prone positioning, and continuous drainage, 91.8% of patients with CSF leakage or dural tears were successfully managed. The most severe complication of TOLF surgery is postoperative paraplegia caused by spinal cord injury. In the study by Li et al. (2016), four patients experienced late postoperative neurological deterioration or persistent worsening paralysis, leading to catastrophic outcomes. Therefore, early diagnosis and treatment are crucial for TOLF patients to achieve better surgical outcomes and minimize complications.

## 5 Pathogenesis of TOLF

Currently, the pathogenesis of TOLF remains unclear. The occurrence of OLF has been reported to be associated with various factors such as mechanical factors, genetic factors, endocrine, and trace elements, and none of these single factors can fully explain its pathogenesis (Kuh et al., 2006). Here we summarize the related research on TOLF in recent years, aiming to provide new insights for the diagnosis and treatment of TOLF.

### 5.1 Mechanical stress

Many studies have shown that mechanical stress plays an important role in the occurrence and development of TOLF. Maigne et al. conducted a study on the ossification of the attachment site of the ligamentum flavum, and found that the rotation amplitude of the zygapophyseal joint was positively correlated with the frequency and size of ossification, indicating that rotational mechanical stress could promote the occurrence and development of TOLF (Maigne et al., 1992).

Fan et al. induced stretch on TOLF cells and found that the expressions of RUNX2, OSX, and ALP increased, suggesting that mechanical stress can could induce osteogenic differentiation of ligamentum flavum cells and thus promote TOLF (Fan et al., 2007a). From the cytological perspective, it was found that OSX was the molecular link between mechanical stress and ossification differentiation.

Cai et al. further discovered that cyclic tensile stress on OLF cells can activate their ossification through the β-catenin signaling pathway (Cai et al., 2012). Ning et al. applied cyclic mechanical stress to primary cells derived from patients with single- and multi-level TOLF, respectively, and found that the mRNA levels of osteogenesis markers were upregulated (Ning et al., 2017). Specifically, the ALP, OPN, BMP2 and OSX mRNA levels were significantly higher in the multiple-level TOLF group, and they increased with the prolongation of stress duration.

Despite relatively numerous studies on mechanical stress, it is still unable to explain the pathogenic mechanism of all cases, and there are few reports about TOLF caused by mechanical stress in Europe, America, Africa, and other regions. The abnormal influence of local spinal stress has its limitations in explaining the pathogenic mechanism of TOLF.

### 5.2 Genetic susceptibility genes

TOLF primarily occurs in Asian populations, and some patients exhibit a familial tendency, indicating that TOLF may be genetically related. Studies have reported that susceptible genes play a significant role in OLF (Qu et al., 2017a; Kong et al., 2007). Single nucleotide polymorphisms (SNPs) have been an important method for studying susceptible genes and genetic factors of diseases due to their widespread distribution in the human genome, strong genetic stability, representativeness, ease of genotyping, and rapid automated analysis (Elkon and Agami, 2017; Park et al., 2019).

#### 5.2.1 RUNX2

Runx2 is an important factor necessary for osteoblast differentiation. It regulates the osteoblast differentiation into osteoblasts, promotes the bone formation and reconstruction, and simultaneously promotes the differentiation of pluripotent stem cells into chondrocytes. One study found that two loci of RUNX2, RS1321075 and RS12333172, were different between OLF patients and the control group, and one haplotype locus was associated with an increased incidence of OLF (Liu et al., 2010). When ligament cells were induced by osteoblast medium, RUNX2 expression was enhanced, while cartilage-forming factors such as connective tissue growth factor, cartilage oligomeric matrix protein, and angiopoietin 1 were significantly increased. After adding RUNX2 siRNA, these gene expressions were inhibited; however, no such changes were observed in the non-ossifying control group cells, suggesting that RUNX2 is involved in ectopic ossification (Kishiya et al., 2008).

#### 5.2.2 BMPs

Bone morphogenetic proteins (BMPs) are multifunctional growth factors that play a crucial role in bone formation. It has been found that BMPR ligands BMP-2/-4 and osteogenic protein-1 (OP-1)/BMP-7 coexist in OLF patients (Hayashi et al., 1997). Moreover, the expression profiles of BMPs and BMPRs in OLF patients are entirely different from those in the control group, indicating that BMPs may be involved in promoting bone formation at the ectopic ossification sites of OLF. Moon et al. found that BMP-2 could significantly upregulate the expression of osteogenic phenotypes, induce the formation of new bone, and promote spinal fusion in human ligamentum flavum cells (Moon et al., 2004). Qu et al. discovered two missense variants of BMP-2 in TOLF (Qu et al., 2017a), namely, c.460C>G:*p*. (R154G) and c.584G>T:*p*. (R195M). Functional analysis showed that the mutant BMP-2 expression promoted osteogenic marker gene expression and osteogenic differentiation, suggesting that genetic factors are involved in TOLF, and the upregulation of BMP-2 expression caused by single-nucleotide substitution may be a possible mechanism of TOLF. Tuo et al. used bioinformatics analysis to identify six BMP-related hub genes, ADIPOQ, *SCD*, *SCX*, *RPS18*, *WDR82*, and *SPON1* in TOLF (Tuo et al., 2023). Through experimental verification, these genes in the experimental group showed differential expression compared with the control group, indicating that they could be potential therapeutic targets for the treatment of TOLF patients.

#### 5.2.3 COL6A1

Type Ⅵ collagen is a kind of fibrillar protein that exists in the extracellular matrix with cytokine activity. It can promote cell differentiation, proliferation, migration, *etc.* It is a marker of fibrous cartilage differentiation and is closely related to osteogenic differentiation (Tanaka et al., 2003). Type Ⅵ collagen protein is composed of three chains, α1, α2, and α3, among which the COL6A1 gene encoding α1 is considered as a susceptible gene for ossification of the posterior longitudinal ligament (OPLL). Ossification of ligamentum flavum (OLF) and OPLL are similar in epidemiology, pathology, and etiology, so COL6A1 is likely to be a potential susceptible gene for OLF (Tanaka et al., 2003; Wang et al., 2019). It was found that the allelic frequency of COL6A1 promoter (−572 T) SNP showed significant differences between OLF patients and normal people (Kong et al., 2007). In addition, there are also significant differences in the total frequency of haplotypes composed of promoter (−572), intron 33 (+20), and intron 32 (−29) SNPs between OLF patients and controls, indicating that COL6A1 may be a susceptible gene for Han Chinese OLF. However, some scholars have drawn opposite conclusions and found that the SNP polymorphism site of COL6A1 is not significantly associated with OLF (Liu et al., 2010), which may be related to locus selection, case selection, and differences in research methods.

#### 5.2.4 FGFs

Fibroblast growth factors (FGFs) and their receptors FGFRs play a significant role in bone development, regulating osteoblast proliferation, differentiation, and apoptosis. FGF2 is an important regulator for bone and cartilage differentiation (Ornitz and Marie, 2015). A comparative study was conducted on the relationship between FGF2, FGFR1, FGFR2, and SNPs in patients and controls using direct sequencing, and the results found that the rs 1,476,217 polymorphism site of FGF2 is associated with OLF (Jun and Kim, 2012). The study of the correlation between genetic polymorphism SNPs and OLF helps to discover OLF susceptibility genes. However, current research on OLF susceptibility genes is still in the exploratory stage. There are certain differences in the results of SNP correlation research and analysis of the same gene and the same site among different scholars. Therefore, conducting large-sample studies is conducive to discovering OLF susceptibility gene sites.

### 5.3 Endocrine and metabolism

Endocrine and metabolic abnormalities may be associated with TOLF. Reports have shown that subjects with TOLF tend to have a higher BMI index (BMI) (Zhang et al., 2022; Zhao et al., 2022). Fan et al. also pointed out that obesity and leptin are risk factors for TOLF (Fan et al., 2007b). Leptin is a peptide hormone secreted by adipose tissue that participates in the metabolism of glucose, fat and energy in the body as well as bone formation. Hyperleptinemia is a common feature of obese individuals. Their study confirmed that leptin participates in the osteogenic differentiation of TOLF cells through the STAT3 signaling pathway. Additionally, patients with conditions such as diabetes, hypertension, and hyperinsulinemia may also develop ligament ossification (Shirakura et al., 2000; Oshima et al., 2020; Koike et al., 2023). Although endocrine and metabolic abnormalities may cause TOLF, there are relatively few research reports. Larger number of cases are needed for further research.

### 5.4 Trace elements

Abnormalities in trace elements may be related to TOLF. It has been reported that fluorosis can lead to the ossification of thoracic ligamentum flavum and other ligaments (Wang et al., 2007; Muthukumar, 2005). Wang et al. performed quantitative analysis of trace elements extracted from ossified tissues of patients with TOLF and found that the content of Ca, F, and Cu increased while the content of Zn, Mn, and Mo decreased (Wang et al., 2008). This indicates that these trace elements may be involved in the ossification process. However, the specific mechanism of these trace elements effects on TOLF remains unclear.

### 5.5 Inflammatory factors

The impact of inflammatory processes on new bone formation has received increasing attention. A tightly controlled inflammatory phase is observed after fractures, which triggers a repair cascade reaction crucial to bone reconstruction (Claes et al., 2012; Schmidt-Bleek et al., 2014). Transgenic animal models have shown the significant role of tumor necrosis factor α (TNF-α) in fracture healing (Gerstenfeld et al., 2003). This indicates that TNF-α participates in promoting postnatal fracture repair, and the development of bone tissue and postnatal repair processes are highly regulated by different mechanisms. Ectopic ossification and some diseases of excessive bone formation occur before the inflammatory phase (Lories and Schett, 2012). Studies have shown that the inflammatory factor Cox2 is involved in the regulation of TNF-α (Xing et al., 2015). However, the impact of inflammatory factors on TOLF remains unclear.

In our recent study, we utilized iTRAQ-labeled quantitative proteomics technology to detect proteins in the yellow ligament of TOLF (Wang et al., 2017). Among them, 282 differentially expressed proteins were identified, and functional clustering analysis revealed that ten of these proteins are related to inflammation, including tumor necrosis factor-α (TNF-α) and prostaglandin reductase 1 (PTGR1).

Our further research found that G1/S-specific proteins cyclin D1 and c-Myc were upregulated after primary ligamentum flavum cells were stimulated by TNF-α (Zhang et al., 2017). Concurrently, Bmp2 and Osx were also upregulated. Additionally, the use of ERK inhibitors abolished the ability of TNF-α to activate Osx expression, suggesting that TNF-α activates Osx through the ERK pathway. This indicates that TNF-α may participate in TOLF by regulating cell proliferation through G1/S-specific proteins cyclin D1 and c-Myc, and inducing osteoblast differentiation via OSX. This is the first time that TNF-α has been found to be involved in TOLF.

Our later study found that PTGR1 could stimulate the expression of c-Myc and CyclinD1 genes in primary ligamentum flavum cells, and JNK pathway inhibitor SP600125 eliminated the expression activity of PTGR1 on c-Myc, suggesting that PTGR1 activated the expression of c-Myc through the JNK pathway (Liu et al., 2023). Our new findings indicate that PTGR1 is likely to be involved in the cell proliferation of TOLF.

Moreover, we have discovered a correlation between the inflammatory cytokine IL-6 and TOLF (Huang et al., 2022). ELISA results indicate a significant increase in IL-6 expression in the culture supernatant of primary TOLF cells. IL-6 can promote the expression of osteoblast factors such as RUNX2 and OSX, and concomitantly upregulate the expression of cyclinD1 and c-Myc. Additionally, this study found that IL-6 regulated BMP2 activation through the p38 MAPK pathway.

Another study found that the expression level of interleukin IL-17 A in TOLF tissue was elevated (Lin et al., 2023). Further experiments revealed that IL-17 A secreted by Th17 cells in the ligamentum flavum may promote cell proliferation and osteogenic differentiation by regulating the β-catenin signaling pathway.

These data collectively indicate that inflammatory factors are associated with the pathogenesis of TOLF.

### 5.6 miRNA

miRNAs play a crucial role in a wide range of physiological and pathological processes, serving as an important family of single-stranded non-coding RNAs (Huang et al., 2017). Qu et al. conducted a comprehensive analysis of the role of miR-132–3p in TOLF (Qu et al., 2016a), and their experiments revealed that miR-132–3p was downregulated during osteogenic differentiation of ligamentum flavum cells. Meanwhile, miR-132–3p could target FOXO1, GDF5, and SOX6, resulting in reduced protein expression of these genes. The downregulation of these genes inhibited osteogenic differentiation. Therefore, miR-132–3p inhibits osteogenic differentiation by targeting osteogenic genes such as FOXO1, GDF5, and SOX6. This study uncovered a novel mechanism of miRNA involvement in TOLF.

Qu et al. found that MiR-199b-5p was downregulated and JAG1 was upregulated during the osteogenic differentiation of ligamentum flavum cells, while osteogenic differentiation was reduced when miR-199b-5p was overexpressed (Qu et al., 2017b). Further experiments revealed that JAG1 is a direct target of miR-199b-5p. MiR-199b-5p exerts an inhibitory effect on osteogenic differentiation in ligamentum flavum cells by potentially targeting JAG1.

Yang et al. found that MiR-490–3p was downregulated during osteogenic differentiation of thoracic ligamentum flavum cells (Yang et al., 2018a). Further researches confirmed that miR-490–3p inhibited osteogenic differentiation by directly targeting FOXO1.

Feng et al. found that the expression of miR-29a-5p was decreased and SATB2 was increased in TOLF tissue (Feng et al., 2020). Further experiments showed that miR-29a-5p was able to effectively inhibit osteoblastic differentiation of cells by targeting SATB2, thereby reducing SIRT1 expression and Smad3 acetylation.

### 5.7 Other factors

Some scholars discovered that Connexin 43 (CX43) was highly expressed during TOLF (Chen et al., 2022). Further experiments showed that CX43 could promote TOLF, potentially by activating the p38 MAPK pathway to regulate RUNX2. Additionally, RUNX2 could bind to the CX43 promoter, forming a positive feedback regulatory loop during TOLF. This suggests that CX43 plays a significant role in TOLF through the p38 MAPK-RUNX2 pathway.

Qu et al. have found that JAG1, Notch2, and HES1 are upregulated in TOLF ligament cells during osteogenic differentiation, and Notch2 overexpression promotes osteogenic differentiation of TOLF ligament cells, indicating that Notch is involved in TOLF. Notch may affect osteogenic differentiation of TOLF ligament cells by interacting with Runx2 and Osx (Qu et al., 2016b).

Yang et al. discovered leucine-rich repeat-containing G protein-coupled receptor (LGR) in the osteogenic differentiation of TOLF cells (Yang et al., 2022). Experimental results demonstrated that LGR5 could promote osteogenesis by activating the Wnt signaling pathway.

Table 2 summarizes the key pathogenic mechanisms that have been reported in association with TOLF.

**TABLE 2:** The pathogenesis associated with TOLF.

Pathogenesis	Brief description
Mechanical stress	Repetitive mechanical stress on the thoracic spine promotes the occurrence and development of TOLF.
Genetic factors	The susceptible genes such as Runx2, BMPs, COL6A1, FGFs are involved in TOLF.
Endocrine and metabolism	Endocrine and metabolic abnormalities such as hyperleptinemia, diabetes, hypertension, and hyperinsulinemia may cause TOLF.
Trace elements	Abnormalities in trace elements such as F, Ca, Zn, Mn, Mo may be related to TOLF.
Inflammatory factor	Inflammatory factors such as TNF-α, PTGR1, IL-6, and IL-17 A promote the proliferation or differentiation of osteoblasts, stimulating osteogenic differentiation and bone formation
MicroRNA	MicroRNAs such as miR-132–3p, miR-199b-5p, miR-490–3p, and miR-29a-5p have been shown to effectively inhibit osteogenic differentiation in ligamentum flavum cells
Others	Other factors such as CX43, Notch and LGR5 may play important roles in the development of TOLF.

Note: This table summarizes the key pathogenic mechanisms associated with TOLF.

## 6 Conclusion and prospects

Although there have been relevant reports on TOLF, its pathogenesis is still in the exploratory stage. The relevant factors mentioned above, such as mechanical stress, genetic susceptibility genes, endocrine and trace element metabolism abnormalities, are currently unable to clearly explain the pathogenesis of TOLF. Due to the fact that genetic and molecular changes often occur earlier than imaging pathological manifestations, how to effectively prevent and treat TOLF is still an unresolved medical challenge. The in-depth study of the molecular mechanism of TOLF will provide clues for exploring its etiology and early diagnosis. The differential expression protein mass spectrometry obtained through proteomics technology in the early stage suggests that inflammatory factors may be involved in the occurrence of TOLF. The study found that TNF-α regulates cell proliferation through cyclin D1 and c-Myc, and induces osteoblast differentiation through OSX to participate in TOLF, revealing the involvement of TNF-α in TOLF and its possible mechanism. Proteomics approach enables a comprehensive understanding of the pathological pathogenesis of TOLF at the protein level. On the other hand, the later research on gene expression profiles based on RNA sequencing shows that angiopoietin-2 is involved in the occurrence of TOLF (Yang et al., 2018b). This study also shows that angiopoietin-2 promotes the gene expression of OSX and is an upstream factor of Osx. In summary, TOLF may be a disease involving multiple factors. Considering the involvement and mechanisms of various factors will help to gain a more comprehensive and in-depth understanding of the pathogenesis of TOLF. On the other hand, conducting research on the pathogenic mechanism of TOLF may provide new ideas for finding upstream regulatory factors of Osx and further developing novel drugs to promote new bone formation to treat osteoporosis.
